# A markov model to evaluate hospital readmission

**DOI:** 10.1186/1471-2288-8-23

**Published:** 2008-04-22

**Authors:** Nicola Bartolomeo, Paolo Trerotoli, Annamaria Moretti, Gabriella Serio

**Affiliations:** 1 Department of Biomedical Science and Human Oncology, Chair of Medical Statistics, University of Bari, Italy; 2 Pneumology Unit, Bari University Public Hospital, Italy

## Abstract

**Background:**

The analysis of non-fatal recurring events is frequently found in studies on chronic-degenerative diseases. The aim of this paper is to estimate the probability of readmission of patients with Chronic Obstructive Pulmonary Disease (COPD) or with Respiratory Failure (RF).

**Methods:**

The Repeated hospital admissions of a patient are considered as a Markov Chain. The transitions between the states are estimated using the Nelson-Aalen estimator. The analysis was carried out using the Puglia Region hospital patient discharge database for the years 1998–2005. Patients were selected on the basis of first admission between 01/01/2001 and 31/12/2005 with ICD-9-CM code of COPD or RF as principal and/or secondary diagnosis. For those selected two possible transitions were considered in the case they had the second and third admission with an ICD-9-CM code of COPD or RF as principal diagnosis.

**Results:**

The probability of readmission is increased in patients with a diagnosis of RF (OR = 1.618 in the first transition and 1.279 in the second) and also in those with a diagnosis of COPD or RF as the principal diagnosis at first admission (OR = 1.615 in the first transition and 1.193 in the second). The clinical gravity and the ward from which they were discharged did not significantly influence the probability of readmission.

**Conclusion:**

The time to readmission depends on the gravity of the pathology at onset. In patients with a grave clinical picture, either COPD or Respiratory Failure, when treated and controlled after the first admission, they become minor problems and they are indicated among secondary diagnoses in any further admission.

## Background

The analysis of non-fatal recurring events, for example repeated admissions, is frequently found in studies on chronic-degenerative diseases like obstructive chronic bronchitis. A repeated admission implies that the patient passes from an acute phase to another acute phase or worse. The hospital history of the patient can thus be considered as a "follow-up" [1] and the subject becomes the protagonist of a Markov process at the finished states, whose transitions between states correspond to the occurrence of one or more events of interest.

Multi-state models that exploit the properties of Markov chains are widely used in medical research because they have a methodological framework useful to describe complex outcomes which are dependent on time [2].

The aim of this study is to evaluate the probability of readmission, that is the probability of transition between two states in patients diagnosed with obstructive chronic bronchitis (admission with principal and/or secondary diagnosis ICD-9-CM: "491.20 – Obstructive chronic bronchitis without acute exacerbation", "491.21 – Obstructive chronic bronchitis with acute exacerbation") and respiratory failure ("518.81 – RF").

Chronic Obstructive Pulmonary Disease (COPD) is one of the most pressing health problems internationally such that by 2020 it is forecast to be the third cause of death [3]. It is a disease which progresses slowly and is frequently diagnosed at a relatively late stage. With acute exacerbation the patient needs to be hospitalised, often for prolonged periods [4]. In Italy, with an aging population, hospitalisation for this disease is increasing, and it is now in fourth place for hospital admissions (data 2003) [5]. Because of this high number of admissions and subsequent public health costs, we wished to examine hospital readmission as one of the factors in COPD reaching such a high position for admissions and identify any possible prevention strategies.

The probability of readmission can depend on variables such as demographic characteristics and linked clinical situations and so in order to estimate the probabilities of transition of the stochastic process we used a regression model which takes account of the covariates. A Cox proportional hazards regression model was used to obtain the estimates of the covariates while for the transition probabilities a Nelson-Aalen estimator was used.

## Methods

### Statistical Analysis

The patient hospital admission history was taken to be a finite state Markov chain, that is a stochastic model with two properties:

for each instant of time *t*, for each pair of states i, j and for each finite series of states k_0_,..., k_t - 1 _the probability of an "event" at time *t *+ 1 depends exclusively on the actual state of the process and not on the previous states: this conditioned probability P{X_t + 1 _= j|X_t _= i} is called transition probability at time *t *of the state i at the state of j;

further the transition probability is stationary in time Pr{X_t + 1 _= j|X_t _= i} = P{X_1 _= j|X_0 _= i} = p_ij_

The matrix P = [p_ij_], is the transition matrix.

In our case we presume that the transition also depends on other factors associated with the subject, so we are interested in the estimate of the matrix *P*[*s*, *t*; *Z*_0_], of dimension k × k, of the transition probability of the state h at the time *s *at the state j at time *t*, for a particular covariate vector Z_0_.

The transition from one admission to the next can be of only two states (readmission/not readmission) and in the data there are variables available that we presume to be constant in time, observed at the moment of the discharge prior to the readmission. The risk function, that represents the probability of the transition at time *t *from one state to the next for an individual with a covariate vector Z_i_, is usually assumed to have the form defined by Cox [6]

(1) *λ*(*t*, *Z*_*i*_) = *λ*_0_(*t*) exp (*β*^*T *^*Z*_*i*_)

where *λ*_0_(*t*) is the basic non specific risk function that depends only on t, for an individual with covariate vector Z_i _= (Z_i1. _Z_i2_,.., Z_ip_) = 0; while exp (*β*^*T *^*Z*_*i*_) is the function chosen to express the effect of the covariate on the basic risk. *λ*_0_(*t*) e *β*^T ^= (*β*_1. _*β*_2_,..., *β*_p_) are the regression coefficients associated with the covariate.

In the study of recurring events the generalisation of the Cox model proposed by Andersen e Gill [7] is often used. Their approach models the repeated admissions, for each subject, as separate observations with the risk not influenced by the number of events (admissions) that the individual undergoes and strongly presumes independence between the multiple observations of a person in time.

In the generalisation proposed by Andersen-Gill (1) assumes the following form:

(2) *λ *(*t*, *Z*_*i*_) = *Y*_*i*_(*t*) *λ*_0_(*t*) exp (*β*^*T *^*Z*_*i*_)

where *Y*_*i*_(*t*) is an indicator of risk of the process that can assume values 0 or 1 indicating, when it has the value 1, that the individual is under observation at time *t*. The time in the Andersen-Gill model is defined as the time that runs between one state (admission) and the next.

Once the value $\overset{⌢}{\beta }$ of the parameters is obtained, it is possible to estimate the transition probability matrix that also takes into account the covariates.

Presuming that T_1._..T_i_,...T_m _are discrete times observed for the m transition taken place in the time interval [*s*, *t*], a consistent estimator of *P*[*s*, *t*; *Z*_0_] is:

(3) $$\overset{⌢}{P}[s,t;Z]=\prod_{i=1}^{m}\left(I+\Delta \overset{⌢}{A}(T_{i},Z_{0})\right)$$

where

(4) $$\Delta \overset{⌢}{A}(T_{i},Z_{0})=\frac{J_{h}(T_{i})\Delta N_{hj}(T_{i})\exp({\overset{⌢}{\beta }}^{T}Z_{hj0})}{\sum_{l=1}^{n}Y_{hl}(T_{i})\exp({\overset{⌢}{\beta }}^{T}Z_{hjl})}$$

represents the Nelson-Aalen estimator calculated on discrete times and corresponds the covariate vector *Z*_*hj*0_, I is the identity matrix of dimension *k *× *k *[8-10].

Let Δ*H*_*kj *_= *N*_*j *_- *N*_*k*_, in (4) *J*_*h *_(*T*_*i*_) Δ*N*_*hj *_(*T*_*i*_) represent the number of subjects that at time *i *make the transition from the state h to the state j; *Z*_*hj*0 _represents the covariate vector of a typical subject; *Y*_*hj *_(*T*_*i*_) and *Z*_*hjl *_respectively indicate the subject that is at state h before the time T_i _and his covariate vector.

Taking into account that the Cox model and its generalisations is defined at proportional risk and namely that the risk relationship in the different groups must remain proportional to the different times, that is it must not vary in time, only those explicative variables that respect the conditions of proportionality have been included in the model. These conditions have been verified with the Log Rank test and where necessary with the Wilcoxon test [11].

Once that the probability transition had been estimated a comparison was carried out between the various typologies of patients with COPD, each with a demographic and clinical covariate vector given upon entering the study.

**Figure 1:**
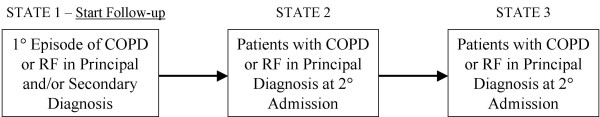
Markov Model at three "states" for patients with Chronic Obstructive Pulmonary Disease.

The model applied to the data of the subjects with COPD was a three state Markov chain with two possible transitions, from the first to second admission and from the second to the third; the third admission was taken as an absorption state (the probability of a patient to remain in this state is equal to 1) (Figure 1).

Admission episodes following the third admission (representing about 15% of the total number of repeated readmissions, Table 1) have not been included in the model because of their low number and because their inclusion would have made the Andersen-Gill model unstable.

**Table 1:** Number of records for number of admissions.

Number of admissions	Number of records	Percentage	Cumulative percentage
1	123,162	57.82%	57.82%
2	39,396	18.50%	76.32%
3	18,459	8.67%	84.98%
4	10,225	4.80%	89.78%
5	6,236	2.93%	92.71%
6 or more	15,525	7.29%	100.00%

### Population included in the study

The analysis was carried out using the Puglia Region hospital patient discharge database for the years 1998–2005, selecting those patients with a first admission for COPD or Respiratory Failure, as principal or secondary diagnosis between the dates 01/01/2001 and 31/12/2005, such that this "follow-up" was of four years.

Only those subjects who in the three years previous to the beginning of the observation period did not have an admission with one of the codes for COPD or RF as principal or secondary diagnosis were selected so as to ensure the selection of those patients who had a first admission in the period under analysis. For each patient a variable was created to indicate admissions prior to the start date of the "follow-up" with one of the following diagnoses: Simple chronic bronchitis (491.0), Mucopurulent chronic bronchitis (491.1), Other chronic bronchitis (491.8), Unspecified chronic bronchitis (491.9), Emphysema (492.x), Asthma (493.x).

To optimise the calculation procedure and to identify a patient typology, the analysis was limited to a sub-group who at the first state of the "follow-up" were of age ≥ 55 (91.48% of admissions for COPD or RF) and who had a first admission for COPD or Acute Respiratory Failure (RF), as principal or secondary diagnosis between the dates 01/01/2001 and 31/12/2005. Those with a second admission within 365 days of the discharge for the first admission, with one of the codes for COPD or RF as principal diagnosis, come into the second state of the Markov chain; the same criteria were adopted between the second and third admissions to identify those who come into the third state.

The variables used within the model were age, sex, anamnesis of chronic respiratory disease previous to 2001, the presence of COPD or RF as principal diagnosis at the start date, the gravity of the disease as indicated by ICD-9-CM of COPD (491.20 without acute exacerbation, 491.21 with acute exacerbation) and acute respiratory failure (518.81), the Charlson index for the gravity of the condition, the presence of other comorbidities not included in the Charlson index, the type of ward and type of hospital at discharge.

The Charlson index, developed in 1987 [12] and adapted to health data banks by Deyo et al. [13], is based on ICD-9-CM diagnosis codes and contains 17 categories of comorbidity, each with an associated weight of from 1 to 6; the overall comorbidity score takes into account both the number of comorbidities and their gravity. In this study the category for COPD has not been considered, it being the morbidity under study.

Because the Charlson index considers the clinical severity without taking into account other pathologies which can influence the probability of readmission, four other covariates were introduced into the model from the presence of at least one of them in the discharge database. These are the ICD-9-CM codes that make up the four supplementary subgroups by additional diagnosis included patients who had at least one ICD-9 coding for the following diseases: Upper respiratory infection (460, 462, 464, 465, 466, 487), Lower respiratory infection (480, 481, 482, 483), Septicaemia (038.x), Heart Failure (428) [14].

The type of ward and the type of hospital at discharge are important factors for the regression model as they indicate how the disease was managed and so are potentially influential on the probability of readmission.

The variables excepting those of age and the Charlson index were made dicotomic. The variable *time *corresponds to the number of days between an admission and the next and represents the risk interval in which the transition can happen. For those admissions on the same day as discharge to the same ward or to a different one or even to a different hospital the time interval has been taken as 1.

## Results

The number of patients aged ≥ 55 selected for the "follow-up" were 123,162 of which 27,550 (22.37%) were readmitted within 365 days. For 33.53% (9,238/27,550) of them the second admission was with one of the three ICD-9-CM codes for COPD or RF as the principal diagnosis. Of these 36.38% had a third admission (3,361/9,238) and of these last there were 1,935 (57.57%) with COPD or RF as the principal diagnosis and so with the transition from the second to the third state of the Markov chain.

In Table 2 are shown the characteristics of the three groups which make up the three states: at the beginning of the "follow-up", after the first transition and after the second. The patients are mainly male over 70 years of age. The percentage of patients with COPD or RF as the principal diagnosis increases passing from the first to third admission. In the beginning the prevalent diagnosis is obstructive chronic bronchitis without mention of acute exacerbation while at second and third admission the prevalent diagnoses are RF and especially obstructive chronic bronchitis with acute exacerbation. The Charlson index score is quite low in all three states, and there is a low frequency of the other comorbidities considered. The first discharge is prevalently from Internal Medicine; while in the second and third there is increasingly a discharge from Pneumology. At the same time the number of local hospitals diminished with a respective increase in specialised hospitals.

**Table 2:** Patient characteristics at start of "Follow-up", after the first transition and after the second transition.

Variable	Start of "Follow-up" N° = 123,162	Transition 1°–2° State N° = 9,238	Transition 2°–3° State N° = 1,935
	
	Percentage or Mean (SD)		
Age at start of "follow-up"	74.36 ± 9.11	74.43 ± 8.60	74.00 ± 8.24
Sex (Male)	62.97	69.18	74.16
Anamnesis Chronic Resp. Disease	14.32	28.21	37.42
COPD in Principal Diagn. al 1° Admiss.	32.19	59.42	68.22
Specific Diagnosis			
491.20	54.88	35.32	21.45
492.21	35.77	53.64	66,46
518.81	16.17	26.86	44,50
Charlson Index	0.65 ± 0.97	0.56 ± 0.88	0.54 ± 0.86
Group of comorbities			
Upper resp. Tract infection	0.28	0.27	0,26
Lower resp. Tract infection	0.39	0.65	1.19
Septicaemia	0.16	0.03	-
Heart Failure	3.07	3.27	3,82
Discharge ward			
Intensive Care	4.69	4.09	4,86
Recovery and Rehabilitation	2.42	5.61	11.11
Internal Medicine	35.53	41.43	35.71
Important others *	43.41	23.31	10.59
Pneumology	13.75	25.49	37.67
University-Research Hospitals	31.32	23.42	19.79

*Medical or Surgical

For the dicotomic variables the presumption of the proportionality of the risk necessary for the correct use of the Cox regression model was checked. From this check, using the Log-Rank test, there was a lack of proportionality of the relative risk functions to the variables which indicate the presence of "Upper respiratory tract infections" (p > 0,05) and of "Septicaemia" (p > 0,05), for this reason they were not used in the regression model.

For the variables sex, heart failure, anamnesis of chronic respiratory disease, COPD or RF as prime diagnosis at first admission, diagnosis "491.21", diagnosis "518.81", discharge ward Intensive Care, or Recovery and Rehabilitation, or Pneumology, or other medical and surgery, lower respiratory tract infections, and hospital type the presumption of proportionality was found to be valid (p = 0,050 for sex, p = 0.035 for heart failure and p < 0,001 for the others).

The factor of discharge from an Internal Medicine ward was inserted into the model in that an admission to this ward more frequently causes a shorter readmission time and so, in this case, for the validity of the proportionality, the value supplied by the Wilcoxon test (p = 0,0129) seemed more appropriate in that it attributes a greater weight to the differences between the functions of probability of readmission at the beginning of the process.

In Table 3 there are the values of each parameter, its significance, and the odds ratio. Age and sex are not significant on the probability of admission for both the second and third times. Neither is the ward type at discharge significant for both changes of state except for Intensive Care that shows a significant increase in the risk of admission for a second time of 168.8%. The Charlson index shows a non-positive influence in the risk of readmission in both the first and second transition. A significant increase of readmission risk for a second admission is shown by having had an anamnesis of chronic respiratory disease before the "follow-up" start date (risk increase of 9.7%) and especially a diagnosis of COPD or RF as prime diagnosis at first admission (risk increase of 61.5%). For the passage from second to third state an anamnesis of chronic respiratory disease is no longer significant, while a diagnosis of COPD or RF as prime diagnosis at first admission remains significant even if the risk increase is less than in the first transition (19.3%). In the passage from first to second state the specific diagnoses of COPD "491.21" and RF "518.81" significantly increase the risk of readmission compared to those with a diagnosis "491.20", 18.7% and 61.8% respectively. In the second transition only the covariate relative to the diagnosis "Acute respiratory failure" remained significant continuing to increase the risk of readmission by 27.9%. The non significance of discharge from Intensive Care in the second transition is probably due their higher mortality and so lack of participation in the transition to the third admission. This is in part confirmed by the analysis of the discharge mode at second admission which shows that 42.67% (198/464) of the patients discharged from Intensive Care are discharged "died" while other wards show a discharge mortality of only 2.06% (181/8,774). The type of hospital where the patient is treated is significant only in the first transition, giving an increased risk of readmission of 13.2% for those discharged from non-university-research hospitals.

**Table 3:** Andersen-Gil Model estimated for patients with COPD.

Variable	Estimated model for the first transition					Estimated model for the second transition				
	
	Value parameter	Pr > |*χ*^2^|	Odds Ratio	CI 95%		Value parameter	Pr > |*χ*^2^|	Odds Ratio	CI 95%	
Age	-0.00027	0.8311	1.000	0.997	1.002	-0.00283	0.3211	1.003	0.997	1.008
Sex (ref. Female)	-0.01819	0.4257	0.982	0.939	1.027	0.00668	0.8992	1.007	0.908	1.116
Anamnesis of Chronic Respiratory Disease	0.09281	**<.0001**	1.097	1.048	1.149	0.04916	0.3063	1.050	0.956	1.154
COPD in Prime Diagnosis at first admission	0.47950	**<.0001**	1.615	1.536	1.699	0.17669	**0.0004**	1.193	1.083	1.315
Diagn. 491.21 (ref. 491.20)	0.17112	**<.0001**	1.187	1.132	1.244	-0.06166	0.2537	0.940	0.846	1.045
Diagn. 518.81 (ref. 491.20)	0.48090	**<.0001**	1.618	1.536	1.703	0.24576	**<.0001**	1.279	1.156	1.414
Charlson index	-0.03458	**0.0058**	0.966	0.943	0.990	-0.03496	0.2311	0.966	0.912	1.023
Infection Lower resp. tract.	-0.17754	0.1773	0.837	0.647	1.084	0.56963	**0.0075**	1.768	1.164	2.684
Heart Failure	-0.00233	0.9693	0.998	0.886	1.123	-0.09379	0.4532	0.910	0.713	1.163
Discharge Intensive Care	0.98873	**0.0096**	2.688	1.271	5.682	1.32490	0.1890	3.762	0.521	27.159
Discharge. Recovery and Rehabilitation	0.28495	0.4546	1.330	0.630	2.806	0.74241	0.4600	2.101	0.293	15.055
Discharge Internal Medicine	0.16840	0.6566	1.183	0.563	2.486	0.97456	0.3313	2.650	0.371	18.932
Discharge Important other	0.00887	0.9813	1.009	0.480	2.120	0.79674	0.4277	2.218	0.310	15.886
Discharge Pneumology	0.21459	0.5698	1.240	0.590	2.608	0.97633	0.3304	2.655	0.372	18.961
Hospital type (ref. U/R Hospital)	0.12407	**<.0001**	1.132	1.076	1.191	-0.03918	0.5182	0.962	0.854	1.083

**Figure 2:**
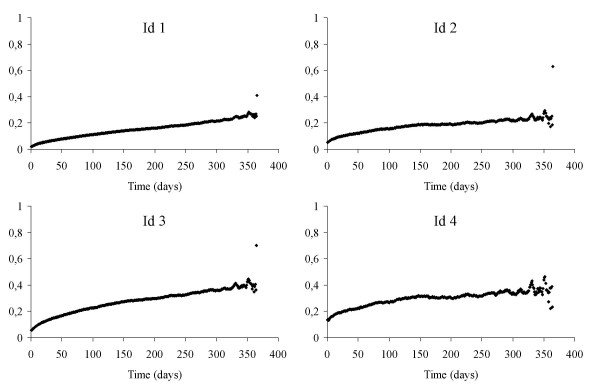
Transition Probability between the first and second recovery for four subjects entered in the follow-up (Id 1 and Id 3 are in less serious condition, Id 2 and Id 4 are in more serious condition).

**Figure 3:**
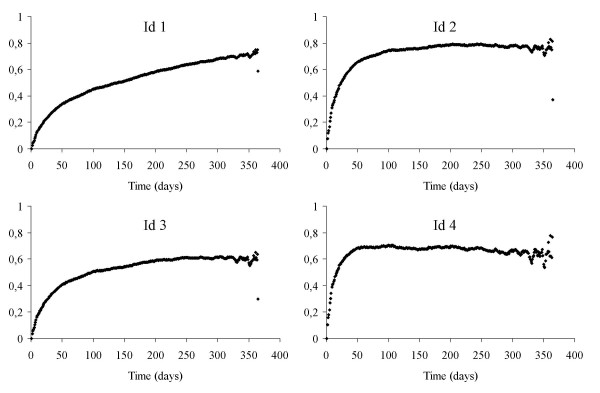
Transition Probability between the second and third recovery for four subjects entered in the follow-up (Id 1 and Id 3 are in less serious condition, Id 2 and Id 4 are in more serious condition).

Figure 2 shows the probability over time of transition from first to second admission and Figure 3 shows it for transition from second to third admission for patients with the same demographic characteristics (male aged 74), the same severity (Charlson index score 0.5) but with four different typologies at the start of the "follow-up" each composed of a combination of two of four different factors. The first factor (A) is entry to the study with a secondary diagnosis of ICD-9-CM 491.20 without specific comorbidities (lower respiratory tract infection or heart failure), without having had admissions for diseases correlated to COPD or RF prior to the period under observation.

The second factor (B) is entry to the study with a prime diagnosis of acute respiratory failure and a secondary diagnosis of lower respiratory tract infection and an anamnesis of chronic respiratory disease.

The third factor (C) is a discharge from the Pneumology ward of an local hospital.

The fourth factor (D) is a discharge from the Intensive Care ward of any non-local hospital.

The first subject Id 1 has factors A and C. The second subject Id 2 has factors B and C. The third subject Id 3 has factors A and D. The fourth subject Id 4 has factors B and D.

The probability of transition from the first to the second state is constantly higher in subjects Id 3 and Id 4 compared to Id 1 and Id 2 with a risk ratio over 1.5 (Table 4A) The trend for all is almost linear. In both typologies C and D the probability of readmission of the more serious subjects of factor B, Id 2 and Id 4, compared to those of factor A, Id 1 and Id 3, was higher in the first six months dropping low or even to null (RR = 1) in the six months following. (After 30 days from discharge Id 2/Id 1 RR = 1.7 and Id 4/Id 3 RR = 1.48.)

**Table 4:** Transition Probability and Relative Risk at 30, 90, 180 and 270 days from previous admission.

Patient	**A**. 1° → 2° Admission				**B**. 2° → 3° Admission			
	
	30 days	90 days	180 days	270 days	30 days	90 days	180 days	270 days
	Transition Probability				Transition Probability			
Id 1	6.3%	10.7%	15.3%	20.0%	25.4%	42.9%	55.8%	65.2%
Id 2	10.7%	15.3%	18.7%	21.6%	54.7%	72.8%	78.1%	77.9%
Id 3	13.9%	21.8%	28.8%	34.5%	31.7%	48.7%	57.5%	60.8%
Id 4	20.6%	26.7%	30.1%	34.0%	60.8%	69.7%	69.5%	66.0%
	Relative Risk				Relative Risk			
Id 2/Id 1	1.70	1.43	1.22	1.08	2.15	1.70	1.40	1.19
Id 4/Id 3	1.48	1.23	1.04	0.98	1.91	1.43	1.21	1.08
Id 3/Id 1	2.20	2.04	1.88	1.73	1.25	1.14	1.03	0.93
Id 4/Id 2	1.92	1.75	1.61	1.57	1.11	0.96	0.89	0.85

The transition from the second to the third state shows higher values than in the first transition as can be seen from the relative risks for both Id 2/Id 1 and Id 4/Id 3, constantly higher in the first 180 days. As in the first transition the second transition showed a higher risk for those treated in Pneumology (Id 2/Id 1) rather than in Intensive Care (Id 4/Id 3). Comparing Id 3 with Id 1 and Id 4 with Id 2 shows the relationship between the types of patient treatment. Each of these over time assumed values lower than 1.00, showing that the ward/hospital type has no influence on the probability of a third admission.

## Discussion

The aim of this study was to evaluate the recurrence of hospitalisation for COPD using the information available in the Puglia patient discharge database so as to determine which characteristics can give an increase in risk of readmission to hospital over time.

Time in hospital prior to entry in the "follow-up", with a diagnosis correlated to COPD or RF are influential only on the probability of a second admission.

A principal diagnosis of COPD or RF ("491.20", "491.21" or "518.81") at entry into the "follow-up", is a strong predictive factor for the probability of readmission, increasing it. A specific principal or secondary diagnosis of COPD or RF has a discriminating effect on the probability of transition to a second admission, remarkably increasing it in patients with Acute Respiratory Failure and in a lesser way in those with Obstructive chronic bronchitis with acute exacerbation.

In the passage from second to third admission, the probability is influenced mainly by the time variable which ameliorates the significance of the covariate compared to the first transition. The significant factors for the probability of a third admission give a lower increase in risk than for the first transition. A discharge from an Intensive Care ward produces contradictory effects, in time, on the probability of a third admission; this could be due to a greater mortality associated with this ward, given the greater severity of patients admitted to this ward; for this it would be useful carry out a record linkage between the Death Register and the Discharge forms, unfortunately the different records are not yet date aligned. In general however the discharge ward does not have significance in the probability of readmission. This is probably due to the ward not being a real variable for the subject under analysis but a variable of the hospital organisation; thus it would be good idea to utilise a multivariate hierarchical model to estimate the coefficients where the type of hospital could be inserted. There could be dependence on the observations inside the second level unit [15] which could be the hospital or the type of ward.

The admission history here analysed (COPD or RF as principal or secondary diagnosis at the second and third admission), characterised by a high incidence of non-acute COPD shown in prime diagnosis at the start of the "follow-up", allows us to hypothesise on the non significance or slight relevance of the Charlson index on the probability or readmission. In fact, in the case where a patient has a more severe clinical picture, as shown by the presence of at least one of the categories of the Charlson index, a successive readmission can be more probable for one of the categories in the index rather than for COPD or RF. In fact COPD or RF, treated and checked thanks to the first admission, become minor problems and in any further admission they are indicated among the secondary diagnoses and are not considered as possible events for successive transitions.

Furthermore, previous studies on mortality after hospitalisation for COPD have shown a higher rate of mortality among patients with more comorbidities [16]; such patients do not contribute to the transition as foreseen by the stochastic model and so the effect of the Charlson index does not show in the determination of the odds ratio.

Variables entered in the model observed the assumption of proportional risk for the Cox model. This allows us to consider transition probability as time-stationary. Only two variables, septicaemia and acute upper respiratory infection, didn't respect the time-stationary assumption and they have been removed from the model under study because of their low frequency in our sample, although they could be very important risk factors for hospital readmission. In different settings violation of time-stationary transition probability could occur and a different model must be adopted.

The choice to limit analysis to only three states, with the third admission as the absorbing state, gave satisfactory results in our study. Death was preferentially used as the absorbing state when data were available.

The characteristics of patients to be included in this study derived from the need to use administrative databases, as the only information available, to conduct epidemiological evaluation. If the aim was to evaluate the probability of admission-readmission cycle the administrative database could be considered reliable, because the states of the process correspond to the admission event.

In the case of an evaluation leaning towards a more epidemiological aspect of the pathology, even if hospital admission are good data, these data must be considered a proxy of the real process represented by the exacerbation of the pathology.

## Conclusion

The application of the assumptions of the Markov chain to the hospital history of the patients affected by Chronic Bronchitis, permits a clear analysis of the probability that patients with certain determined characteristics will have a new admission to hospital. More, the use of the health region database in multi-state models permits the evaluation of the probabilities of readmission in different scenarios, especially because, as Borg et al. conclude [17], large long-term clinical studies would not be feasible. The method, using the Nelson-Aalen estimator for the probabilities of transition, interprets the data showing it can integrate the effect of time and the other covariates. To estimate the coefficients in the Cox model, a multilevel model should be performed, a model which takes into account that the patients transit inside a hierarchical health system.

## Competing interests

The authors declare that they have no competing interests.

## Authors' contributions

NB defined the theoretical setting, build up software for data analysis, conducted analysis and interpretation of data, drafted the manuscript. PT conceived the study, defined the study case and choose ICD code, proposed risk adjustment methods, contributed to interpretation of data, drafted the manuscript. AM revised the clinical setting, defined the study case, revised the manuscript. GS defined the theoretical setting, contributed to interpretation of data, contributed to draft the manuscript.

## Pre-publication history

The pre-publication history for this paper can be accessed here:


